# The Biodistribution of the Spike Protein after Ad26.COV2.S Vaccination Is Unlikely to Play a Role in Vaccine-Induced Immune Thrombotic Thrombocytopenia

**DOI:** 10.3390/vaccines12050559

**Published:** 2024-05-20

**Authors:** Sonia Marquez-Martinez, Selina Khan, Joan van der Lubbe, Laura Solforosi, Lea M. M. Costes, Ying Choi, Satish Boedhoe, Mieke Verslegers, Marjolein van Heerden, Wendy Roosen, Sandra De Jonghe, Hendy Kristyanto, Veronica Rezelj, Jenny Hendriks, Jan Serroyen, Jeroen Tolboom, Frank Wegmann, Roland C. Zahn

**Affiliations:** 1 Janssen Vaccines & Prevention (JVP), 2333 CN Leiden, The Netherlands; soniamqmz@gmail.com (S.M.-M.);; 2 Janssen Research & Development (JRD), B-2340 Beerse, Belgium

**Keywords:** vaccine, adenovirus, VITT, spike, COVID-19

## Abstract

Ad26.COV2.S vaccination can lead to vaccine-induced immune thrombotic thrombocytopenia (VITT), a rare but severe adverse effect, characterized by thrombocytopenia and thrombosis. The mechanism of VITT induction is unclear and likely multifactorial, potentially including the activation of platelets and endothelial cells mediated by the vaccine-encoded spike protein (S protein). Here, we investigated the biodistribution of the S protein after Ad26.COV2.S dosing in three animal models and in human serum samples. The S protein was transiently present in draining lymph nodes of rabbits after Ad26.COV2.S dosing. The S protein was detected in the serum in all species from 1 day to 21 days after vaccination with Ad26.COV2.S, but it was not detected in platelets, the endothelium lining the blood vessels, or other organs. The S protein S1 and S2 subunits were detected at different ratios and magnitudes after Ad26.COV2.S or COVID-19 mRNA vaccine immunization. However, the S1/S2 ratio did not depend on the Ad26 platform, but on mutation of the furin cleavage site, suggesting that the S1/S2 ratio is not VITT related. Overall, our data suggest that the S-protein biodistribution and kinetics after Ad26.COV2.S dosing are likely not main contributors to the development of VITT, but other S-protein-specific parameters require further investigation.

## 1. Introduction

The Ad26.COV2.S COVID-19 vaccine (JCOVDEN, Johnson & Johnson) has been broadly used in the prevention of severe acute respiratory syndrome coronavirus 2 (SARS-CoV-2) infection and has been shown to elicit protection, lasting 9 months or longer, against severe disease [1].

Ad26.COV2.S is a recombinant and replication-deficient human adenovirus type 26 (Ad26) vector encoding the full-length spike protein (S protein) of SARS-CoV-2 and is based on the Wuhan strain [2,3]. The S protein encoded by this monovalent vaccine contains two stabilizing proline substitutions and mutations in the furin cleavage site to preserve the prefusion conformation [2]. All other COVID-19 vaccines originally licensed in Europe and North America also encode the S protein based on the original SARS-CoV-2 Wuhan strain but do not contain mutations in the wild-type furin cleavage site [4].

Thrombosis with thrombocytopenia syndrome (TTS) has been reported following COVID-19 vaccination, and the term vaccine-induced immune thrombotic thrombocytopenia (VITT) has been used to describe cases that are likely vaccine-related. VITT is a rare but severe adverse event characterized by thrombocytopenia and thrombosis, often in atypical anatomical locations, and the presence of antibodies against platelet factor 4 (PF4) [5]. VITT may be a rare event, but as adenoviral vectors have valuable attributes for use as prophylactic vaccines, understanding the severe rare events associated with use will allow their future use as a vaccine platform. VITT has been reported in approximately 2.3 to 5.5 cases per 1 million vaccinees after Ad26.COV2.S dosing, depending on the definition of the syndrome [6] (e.g., definitions from the Centers for Disease Control and Prevention, USA [7,8]; the Pharmacovigilance Risk Assessment Committee of the European Medicines Agency [9]; and the Brighton Collaboration Case Definition [10]). The estimated incidence of VITT is 8.1 per 1 million vaccinees after the first dose of ChAdOx1 and 2.3 per 1 million vaccinees after the second dose [11]. VITT occurs 5 to 43 days [7,12,13] after the first dose of ChAdOx1 nCoV-19 [14] or Ad26.COV2.S [15]. It has also been reported sporadically after COVID-19 vaccination with mRNA-1273 [16], inactivated COVID-19 [17,18], or Gam-COVID-vac vaccinees [19]. Notably, a case of VITT was recently identified 10 days after Gardasil 9 vaccination for human papillomavirus [20].

The mechanism of induction of VITT has not yet been elucidated, but multiple hypotheses have been proposed. Some hypotheses focus on the role of the adenoviral particle in the development of VITT. It was originally reported that the binding of the adenoviral particle to PF4 could play a role in the development of VITT [21], but recent studies showed no binding between PF4 and Ad26.COV.2.S [22,23]. There is also conflicting literature associating human adenovirus infections with a prothrombotic disorder resembling VITT [24,25]. However, the high prevalence of natural human adenovirus infections without a high frequency of associated prothrombotic disorders, and the fact that TTS-like disease has also been described in patients with COVID-19 [26,27], suggests a multifactorial mechanism for VITT.

Other potential factors influencing the development of VITT include interactions of the S protein with platelets/endothelial cells that might lead to the activation of coagulation pathways [28,29]. The SARS-CoV-2 S protein has been shown to cause vascular damage in hamsters [30] and has been detected within the thrombus and in the adjacent blood vessel wall in VITT patients with cerebral venous thrombosis [31]. Moreover, the S protein may activate coagulation pathways through the binding of angiotensin-converting enzyme 2 (ACE2) directly on platelets and/or endothelial cells [32]. The S protein may also activate platelets and trigger the release of PF4 molecules that activate memory PF4 B-cells [32,33], and the activation of memory PF4 B-cells along with an inflammatory co-stimulus due to the interaction and/or damage to the endothelium could lead to release of high titers of anti-PF4 antibodies and subsequent induction of VITT.

To gain further insight into the potential contribution of the S protein in VITT in the context of an Ad26-based vaccine, it is important to understand the distribution of the S protein, including the S protein expression in platelets and/or endothelial cells or binding, and its composition after Ad26.COV2.S dosing.

To this end, we investigated the biodistribution of the S protein and characterized the circulating S protein after intramuscular (IM) dosing with Ad26.COV2.S in preclinical models and clinical samples.

## 2. Materials and Methods

### 2.1. Ethics Statement

The rabbit study was conducted at JRD Belgium, according to the European guidelines (EU directive on animal testing 86/609/EEC) and Belgian guidelines, and with the principles of euthanasia as stated in the Report of the American Veterinary Medical Association Panel.

The mouse studies were conducted at JVP, according to the European guidelines (EU directive on animal testing 86/609/EEC) and Dutch national legislations, and approved by the Central Authority for Scientific Procedures on Animals (Centrale Commissie Dierproeven).

The nonhuman primate (NHP) study was conducted at Charles River Laboratories (Wilmington, MA, USA), according to the standard operating procedures by technical staff and approved by the Institutional Animal Care and Use Committee (IACUC) at Charles River Laboratories. The test facility is accredited by the American Association for Accreditation of Laboratory Animal Care (AAALAC), and animal experiments were performed in accordance with the standards of the AAALAC International’s reference resource [34,35].

### 2.2. Participants

The levels of S protein were measured by assessment in serum samples from the clinical studies COV1001, COV3003, COV3001, and COV3009 as part of exploratory research. Samples from 5 participants, aged ≥18 to ≤55 years, who were dosed with 5 × 10^10^ vp of Ad26.COV2.S and were enrolled in the study COV1001 (NCT04436276) at the Beth Israel Deaconess Medical Center (Boston, MA, USA) [36], were used for S-protein analysis. The clinical study COV3003 (NCT04908722) was a randomized, double-blind, phase 3 study to evaluate 6 dose levels of Ad26.COV2.S administered as a 2-dose schedule in healthy adults ≥18 to ≤55 years of age. The levels of S protein were measured in participants who enrolled in a substudy and received 1 dose of 9 × 10^10^ vp, 5 × 10^10^ vp, or 1.25 × 10^10^ vp of Ad26.COV2.S.

The serum samples obtained after mRNA COVID-19 vaccination were collected from vaccinees who received the placebo within the clinical studies (COV3001 or NCT04505722 [1] and COV3009 or NCT04614948 [37]) but who self-reported that they received an mRNA-based COVID-19 vaccine outside the clinical studies. Although the participants followed the clinical-study-defined blood sample collection schedule, specific post-mRNA vaccination timepoints were not preplanned. Therefore, serum samples from participants who received mRNA-based COVID-19 vaccines outside the clinical studies were collected at different post-mRNA vaccination timepoints.

All clinical study protocols were conducted following the Declaration of Helsinki and International Council for Harmonisation Good Clinical Practice Guidelines (ICH-GCP) and were approved by both local and national independent ethics committees, as well as institutional review boards (IRBs). All participants provided informed consent.

### 2.3. Vaccines

**Figure A1:**
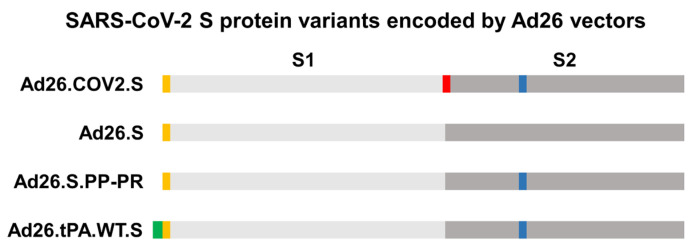
Ad26 vectors encoding variants of the SARS-CoV-2 S protein. From top to bottom: (1) full-length S in which furin cleavage site mutations and proline substitutions have been introduced, (2) native full-length S, (3) full-length S with only the proline substitutions, and (4) full-length S in which tissue plasminogen activator signal peptide is added upstream of the wild-type signal peptide. Green bar represents tissue plasminogen activator signal peptide, yellow bars represent wild-type signal peptide, red vertical lines represent furin cleavage site mutations, and blue vertical lines represent proline substitutions.

Replication-incompetent, E1/E3-deleted recombinant Ad26 vectors were engineered using the AdVac^®^ system as described elsewhere [38,39], with Ad26 encoding different versions of the SARS-CoV2 S protein from the SARS-CoV-2 isolate Wuhan-Hu-1 (GenBank accession number: MN908947). The constructs encoded a native full-length spike protein (Ad26.S) or one in which proline substitutions (K986P, V987P) were introduced (Ad26.S.PP-PR), a full-length S protein in which the furin cleavage site was abolished by the amino acid changes R682S and R685G and proline substitutions (K986P, V987P) were introduced (Ad26.COV2.S), and a native full-length S protein with a tissue plasminogen activator (tPA) sequence upstream of the S protein (Ad26.S.tPA.WT.S) (Figure A1) [2]. The Ad26 vector Ad26.ZIKV.001 (encoding Zika virus envelope protein) was used as a control [38,40]. The Ad26-mediated expression of the various transgenes was confirmed by Western blot analysis of cell-culture lysates from infected A549 cells or by polymerase chain reaction.

BNT162b2 mRNA (Comirnaty, Pfizer/BioNTech) [41] was used in Figures 4 and 5 to compare the kinetics of expression and composition of the S protein with Ad26.COV2.S. BNT162b2 mRNA_encodes a wild-type prefusion stabilized SARS-CoV2 S protein with no mutations in the furin cleavage site.

### 2.4. Purified SARS-CoV-2 S Protein and Adjuvant 

The S protein used for dosing (COR201225) contains amino acids 14-1208 of the Wuhan-Hu-1 SARS-CoV-2 S protein (GenBank accession no. MN908947) and includes the stabilizing mutations R682S, R685G, N532P, T572I, D614G, G880C, F888C, A944P, and V987P as described elsewhere [42]. The protein was produced in Exp293F cells and purified by a 2-step purification protocol by first applying cleared culture supernatant on a Galantus nivalis-lectin column (Vectorlabs, AL-1243, Newark, CA, USA) with 40 mM Tris, 500 mM NaCl pH 7.4 as a buffer. Elution was performed with the same buffer with additional 1 M Mannopyranoside to a final pH of 7.4. Eluted protein was concentrated and subsequently loaded on a Superdex200 Increase column (GE HealthCare, Chicago, IL, USA) in 20 mM Tris, 150 mM NaCl pH 7.4 as a buffer. Sucrose was added to a final concentration of 5% before snap freezing in liquid nitrogen. Protein was tested for bioburden and endotoxin levels (Endosafe nexgen-PTS test, Charles River, Needham, Wilmington, MA, USA) before use. Aluminum hydroxide Al(OH)_3_ was produced from Alhydrogel 2% (InvivoGen, Toulouse, France) at JVP. The COR201225 protein and adjuvant were mixed by pipetting and incubation on a roller-bench for 1 h at room temperature (RT) before dosing. The S protein used in the cell lysate in vitro test (COR200672) contains amino acids 14-1208 of the Wuhan-Hu-1 SARS-CoV-2 spike (GenBank accession no. MN908947) and similar stabilizing substitutions to COR201225 (R682S, R685G, N532P, T572I, D614N, G880C, F888C, A942P, K986P, and V987P). The protein was produced and purified according to the same protocol as COR201225 described above.

### 2.5. Animals and Housing

A total of 40, 14-week old, healthy male New Zealand White (NZW) rabbits (body weight 2.4–3.4 kg at study start) were included. Animals were supplied by Charles River Laboratories (France). Animals were kept in a biosafety level 2 (BSL-2) facility under specific pathogen-free conditions. Animals were single-housed in stainless steel cages placed in study-dedicated rooms.

Female BALB/c and C57BL/6 mice (specific pathogen-free), aged 5 to 12 weeks at the start of the study, were purchased from Charles River Laboratories (Sulzfeld, Germany). Female Jh C57BL/6NTac-Igh-Jem1Tac and C57BL/6 control mice (specific pathogen-free), aged 10 weeks at the start of the study, were purchased from Taconic Biosciences. Animals were kept in a BSL-2 facility under specific pathogen-free conditions. 

Cynomolgus macaques (*Macaca fascicularis*) of Cambodian origin were aged 5.32 to 9.22 years and weighed 5.8 to 8 kg (males) and 3.3 to 4.4 kg (females) at the initiation of dosing. The evaluations were performed in accordance with the standard operating procedures by technical staff.

For all animal studies, animals were kept under controlled, recorded environmental conditions of humidity, temperature, and 12 h light cycle. Animals were provided with sensory and cognitive environmental enrichment including occupational material. Animals were fed a standard diet ad libitum and tap water was provided ad libitum through an automated system. Animal well-being and health surveillance was monitored at least daily by husbandry staff. Preset humane endpoints were used to define study-unrelated sacrifice criteria by a veterinarian. All measures were taken to minimize pain, distress, and suffering, and all procedures were performed by trained personnel.

### 2.6. Animal Study Designs and Procedures

**Figure 1:**
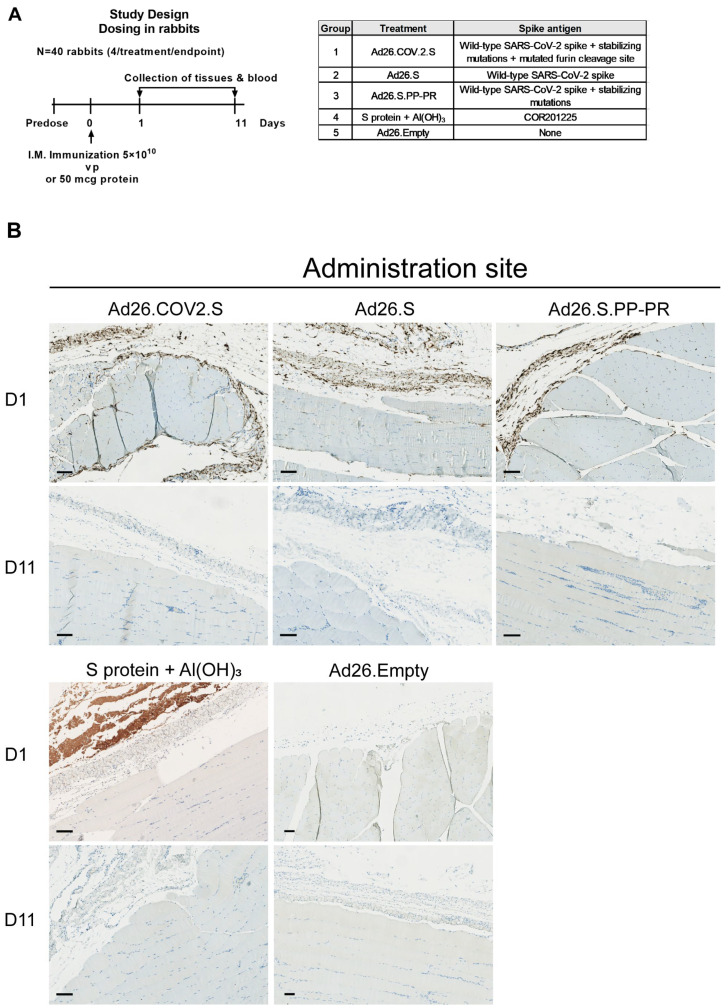

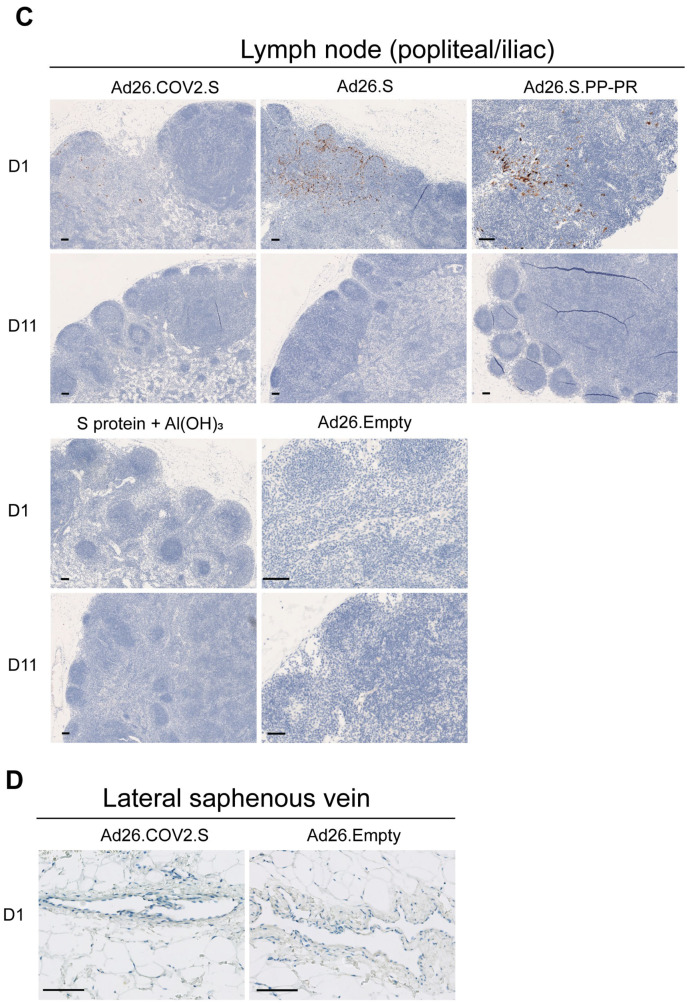
The spike (S) protein was detected at the site of injection and draining lymph nodes, but not in the veins 1 day after intramuscular (IM) Ad26.COV2.S dosing in rabbits. (**A**) Rabbits (*n* = 40) were dosed with 5 × 10^10^ vp of Ad26.COV2.S, Ad26.S, Ad26.S.PP-PR, Ad26.Empty or 50 μg of S protein + Al(OH)_3_. Anti-SARS-CoV2 S1 staining by immunohistochemistry of (**B**) muscle (administration site) and (**C**) draining lymph nodes (popliteal and iliac) at Day 1 (positive) and Day 11 postdosing (negative), and (**D**) lateral saphenous vein at Day 1 postdosing. In brown are the positive areas for the target protein. The black bar equals 100 μm.

In the rabbit study, NZW rabbits were divided into 5 study groups with 8 animals per group. The animals received a single IM dose of 5 × 10^10^ vp (full human dose) of Ad26.COV2.S, Ad26.S (encodes the wild-type SARS-CoV2 S protein [2]) or Ad26.S.PP-PR (encodes the SARS-CoV2 S protein with 2 prolines in the hinge region [2]) (Figure 1A). An IM injection with an Al(OH)_3_ adjuvanted soluble S protein (COR201225) (50 mcg) was included as a positive control and IM injection of an empty Ad26 vector (Ad26.Empty) as a negative control (Figure 1A). All vaccines were administered in a 0.5 mL volume. Blood sampling was performed from the central ear artery. The total blood volume and sampling frequency was performed according to good ethical practices. Minimal to slight erythema at the administration site was noted in the groups (including vehicle groups) receiving an IM injection and was considered to represent the normal, expected reaction related to the IM injection procedure [43]. At the end of the study, animals were anaesthetized by an intravenous injection of pentobarbital and sacrificed by exsanguination via the inguinal blood vessels. Terminal blood sampling was performed via the inguinal blood vessels.

Mice were immunized IM with different doses in 50 μL of vaccine preparation as indicated in the text (Section 3.3 and Section 3.5). Intermediate blood samples were collected via submandibular bleeding at different timepoints (Figure 7 and Figure A4). At the end of each study, mice were anaesthetized with isoflurane, exsanguinated through heart puncture, and sacrificed by cervical dislocation. Blood was processed for serum isolation and spleens were collected for humoral and cellular assays, respectively. Control mice received a buffer solution (15 mM citric acid, 75 mM NaCl, 2-hydroxylpropyl-β-cyclodextrin 5% (*w*/*w*), 0.03% PS-80 pH 6.2).

In the NHP study, 8 cynomolgus macaques (4 females and 4 males) were immunized IM (left thigh) on Days 0 and 56 with 5 × 10^10^ vp/animal (full human dose) of Ad26.COV2.S in 0.5 mL of vaccine preparation. The total blood volume and sampling frequency was performed according to good ethical practices. 

### 2.7. Processing of Whole Blood for Serum and Plasma Generation

Rabbit serum samples were prepared from clotted blood drawn into serum tubes after centrifugation at 1900× *g* for 5 min at RT. Serum was stored at −80 °C until the time of analysis. Rabbit plasma was prepared by drawing whole blood into anticoagulant-containing tubes (ethylenediaminetetraacetic acid (EDTA)) and centrifuging at 2000× *g* for 15 min at 4 °C. The pellet (referred to as the blood cellular fraction) was collected and stored at −80 °C. The supernatant (platelet-depleted plasma) was collected into sterile 15 mL Falcon tubes, mixed by inversion, and stored at −80 °C.

Mouse serum samples were prepared from clotted blood drawn into Eppendorf tubes after centrifugation at 2660× *g* for 4 min followed by 20,800× *g* for 1 min. Serum was stored at −20 °C until the time of analysis.

### 2.8. Detection of S Protein in Tissues by Immunohistochemistry Staining

Rabbit samples from the administration site (skin, muscle—left biceps femoris), draining lymph nodes (iliac and popliteal), and vein (lateral saphenous/cava caudalis) were fixed in 10% formalin and embedded in paraffin and sectioned at 5 μm thickness. Sections of administration site, draining lymph nodes, spleen, and veins were stained immunohistochemically (Ventana Discovery Ultra autostainer, Roche Diagnostics, Pleasanton, CA, USA) using a hapten multimer horseradish peroxidase (HRP)-based technology and diaminobenzidine (DAB) tetrahydrochloride detection method, by a monoclonal S-protein antibody: SARS-CoV-2 Spike S1 Subunit Antibody (clone 1035206, R&D, catalog no. MAB105403) at 5 μg/mL (S1 antibody). The isotype control (Mouse IgG1 Abcam ab18443, Cambridge, UK) and the Ad26.Empty group served as negative controls. The sections were scored semi-quantitatively for S-protein (S1)-immunoreactive cells.

### 2.9. Detection of Spike mRNA in Tissues by RNAscope

Formalin-fixed paraffin embedded blocks from the administration site and lymph nodes were processed at 5 μm thickness for in situ detection of SARS-CoV-2 S mRNA (Advanced Cell Diagnostics (ACD), catalog no. 1116539-C1, Newark, NJ, USA). For this, the RNAscope^®^ VS Universal AP assay for Ventana Discovery Ultra (Advanced Cell Diagnostics, Newark, CA, USA) was used according to the manufacturer’s protocol. Briefly, slides were dewaxed (RNAscope^®^ VS Universal Dewax, Advanced Cell Diagnostics (ACD), Newark, NJ, USA) and subjected to target retrieval (RNAscope^®^ VS Universal Target Retrieval v2) at 97 °C and protease pretreatment (RNAscope^®^ VS Protease) to uncover the target RNA sites. Probe pairs were subsequently hybridized to the target mRNA, cascaded with AMPs 1–7, including AP-labeled probes, according to ACD’s patented signal amplification and background suppression technology. Target RNA was detected using Fast Red as a chromogenic substrate (mRNA RED detection kit, Roche Diagnostics) and punctate red dots representing transcripts were evaluated using a standard brightfield microscope. For each sample, the housekeeping gene peptidylpropyl isomerase B (PPIB) was used as a positive control probe and the *Bacillus subtilis* strain SMY methylglyoxal synthase (mgsA) gene, partial cds dihydrodipicolinate reductase (DapB) gene was used as a negative control probe.

### 2.10. Detection of the S Protein in Blood by Electrochemiluminescence

Complete EDTA-free protease inhibitor (Roche, Basel, Switzerland) was added to plasma samples. Plasma samples were centrifuged for 3 min, 2000× *g* at 4 °C to remove particulates before assay. For the blood cellular fraction, samples were lysed with cold lysing buffer (RIPA buffer; complete EDTA-free protease inhibitor, Roche, 04693116001; benzonase) on ice for 1 h, inverting the tubes every 15 min. Cellular fraction samples were centrifuged for 5 min, 600× *g* at 4 °C to clarify the lysate of cell debris and larger membrane fractions.

The S-PLEX SARS-CoV-2 Spike detection assay (Meso Scale Discovery, Rockville, MD, USA, detecting the presence of the S-protein RBD, direct communication from the manufacturer) was used to detect S protein in the plasma, serum, or blood cellular fraction samples, according to the manufacturer’s instructions, using phosphate-buffered saline (PBS) +0.05% Tween-20 as washing buffer, and all incubation steps were performed at 27 °C. Analysis was performed with the MSD Discovery Workbench version 4.0.

In the experiment presented in Figure 4A, mouse serum was diluted 1/100 for the samples in the Ad26.COV2.S and buffer control groups, and 1/10,000 for the samples in the BNT162b2 group. In Figure A5A, all serum samples were diluted 1/100. All other samples were measured undiluted. In Figure 7B, all serum samples were diluted 1/5.

### 2.11. Detection of S1–S2 Protein in Blood by Electrochemiluminescence

The S-PLEX SARS-CoV-2 Spike detection assay was used to detect S protein containing the S1 and S2 domains (S1–S2) in serum samples by exchanging the anti-RBD capture antibody with an antibody against the conserved S2 stem helix region of the S protein (CC40.08). Briefly, plates were coated overnight with CC40.08 (1.7 μg/mL) in coating reagent and diluent supplied by the assay kit. After coating, the assay was followed as described by the manufacturer (Meso Scale Discovery). The washing buffer was PBS + 0.05% Tween-20, and an automated plate washer was used for the washing steps (washing protocol as described in the kit manual). All incubation steps were performed at RT except for the TURBO-TAG detection solution (27 °C). Analysis was performed with the MSD Discovery Workbench version 4.0.

Mouse serum was diluted 1/2 for the samples in the Ad26.COV2.S and buffer control groups, and 1/10 for the samples in the BNT162b2 group (Figure 4B). In the experiment presented in Figure A5B, serum samples were diluted 1/2.

### 2.12. Detection of Spike-Specific Immunoglobulin G (IgG) in Serum by Enzyme-Linked Immunosorbent Assay (ELISA)

Spike-binding IgG was measured in total mouse serum by ELISA. Briefly, ½ area 96-well OptiPlates (Perkin Elmer, Waltham, MA, USA) were coated with SARS-CoV-2 S protein at 2 μg/mL (COR200153, [44] in 1X PBS, Gibco, ThermoFischer, Waltham, MA, USA) overnight at 4 °C. Remaining S protein was removed and the plates were washed 3 times with PBS + 0.05% Tween-20 (Calbiochem, Merck Millipore, Burlington, MA, USA) (PBS-T) and blocked with PBS 1% casein (ThermoFischer, Waltham, MA, USA) for at least 1 h at RT, followed by another wash. Serum from mice was serially diluted (starting dilution 1:50) in sample buffer (1X PBS/1% Casein). Diluted samples were transferred to the coated Maxisorp 96-well ELISA plates (50 μL/well in total), incubated for 60 min at RT, and washed as described above. Bound IgG was detected with goat-anti-mouse IgG (H + L) conjugated to HRP (KPL/SeraCare, Milford, MA, USA), and detection substrate (electrochemiluminescence [ECL]) was added and incubated for 10 min. Luminescence was read on a BioTek Synergy Neo plate reader and a 4-parameter logistic curve fitting was performed. The final reportable values (log10EC50) are derived from the fitted curve.

Serum from cynomolgus macaques was used to assess IgG binding to SARS-CoV-2 S protein by ELISA using a recombinant soluble S protein based on the Wuhan-Hu-1 SARS-CoV-2 strain (MN908947) and stabilized by 2 point mutations in the S1/S2 junction that knock out the furin cleavage site, and by 2 newly introduced prolines in the hinge region in S2. In addition, the transmembrane and cytoplasmic regions were replaced by a foldon trimerization domain followed by a His-tag, allowing the trimeric protein to be produced and purified as soluble protein. Briefly, 96-well microplates were coated with the S protein for a minimum of 16 h at 4 °C. Plates were washed in PBS/0.05% Tween-20 and blocked with 5% skim milk (BD Life sciences, Franklin Lakes, NJ, USA) in PBS/0.05% Tween-20 for 1 h at RT. Samples were serially diluted starting at 1:50, added to the plates, and incubated for 1 h at RT. After washing, the plates were incubated with peroxidase conjugated goat anti-human IgG (Jackson ImmunoResearch, West Grove, PA, USA) for 1 h at RT, washed again, and developed with tetramethylbenzidine substrate for 30 min at RT. The optical density was read at 450/620 nm and a 4-parameter logistic curve fitting was performed. The antibody titers (expressed in ELISA units [EU]/mL) were determined in relation to the standard based on all dilutions tested. 

Serum samples from human vaccinees were assessed for SARS-CoV-2 S-specific binding antibody concentrations as previously described [3]. In brief, purified SARS-CoV-2 pre-S antigen was adsorbed to the wells of a microplate and diluted serum samples (test samples, standard, and quality controls) were added. Unbound sample was washed away, and enzyme-conjugated anti-human IgG added. After washing the excess conjugate away, 3,3′,5,5′-tetramethylbenzidine colorimetric substrate was added. After the established time period, the reaction was stopped. A reference standard on each test plate was used to quantify the amount of antibodies against SARS-CoV-2 pre-S in the sample according to the unit assigned by the standard (EU/mL).

### 2.13. Serum Coincubation Study

The interference of anti-S-protein antibodies in the detection of S protein was assessed in an in vitro serum coincubation study. Rabbit sera obtained from a different study [45] predose or 28 days postdosing with either Ad26.COV2.S or Ad26.ZIKV001 were mixed with serum obtained 1 day postdosing with Ad26.COV2.S (Figure 1A). Serum samples were mixed in a 1:1 ratio (20 μL + 20 μL). Complete EDTA-free protease inhibitor (Roche, Basel, Switzerland) was added to all samples (including single controls) and samples were shaken at 700 rpm for 3 h at RT. The S-PLEX SARS-CoV-2 Spike detection assay (Meso Scale Discovery, Rockville, MD, USA) was used to determine the concentration of S protein in the samples.

### 2.14. Statistical Analysis 

Data were log-transformed and groups were compared using a 2-sample *t*-test (paired or unpaired) or analysis of variance (ANOVA) in case of non-censored data. For censored data, a Tobit model analysis with correction for multiple comparisons (Bonferroni adjustment) was applied where indicated. *p*-values < 0.05 were considered statistically significant. Correlation coefficients were calculated where indicated using the Spearman rank correlation.

## 3. Results

### 3.1. S Protein and S mRNA Were Detectable at the Site of Administration and in Draining Lymph Nodes at Day 1 but Not Day 11 after IM Ad26.COV2.S Vaccination in Rabbits

To study the biodistribution of the S protein after Ad26-based COVID-19 vaccination and understand the effect of stabilizing mutations of the S protein, rabbits were dosed IM with Ad26 vectors encoding differently stabilized versions of the SARS-CoV-2 S protein and were sacrificed 1 or 11 days after dosing (Figure A1 and Figure 1A).

On Day 1 post IM injection, S protein was present at the administration site (Figure 1B) and in the draining lymph nodes (iliac and/or popliteal) (Figure 1C) of groups dosed with an S-encoding Ad26 vector as evaluated by immunohistochemistry (IHC). At the injection site, membranous/cytoplasmic S protein staining was mainly observed in round to elongated cells (considered macrophages and/or fibroblasts) in connective tissue, while the S protein could not be detected in myocytes at the administration site. In draining lymph nodes, S protein was detected in presumed macrophages and dendritic cells, according to their morphology. The S protein was not detected in arteries/veins (i.e., blood vessels at the injection site, or lateral saphenous vein and vena cava caudalis) (Figure 1D). At Day 11 after the IM injection with Ad26-based vaccines, the S protein was no longer detected in any of the tissues examined (Figure 1).

**Figure A2:**
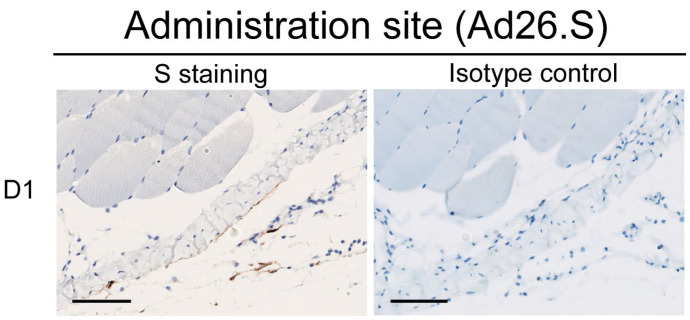
Immunohistochemistry S-protein staining and isotype control in muscle after IM Ad26.S dosing in rabbits. Anti-SARS-CoV2 S1 or isotype control staining by immunohistochemistry of muscle (administration site) at Day 1 post Ad26.S dosing. The black bars represent the magnification of the images (100 μm).

In animals dosed with the recombinant soluble S protein COR201225 (+Al(OH)_3_), the S protein was only detected at the administration site, mainly without cellular association, on Day 1 after an IM injection and it was no longer detected in any of the tissues examined on Day 11. No S-protein signal was detected in the Ad26.Empty group (Figure 1) or the isotype control (Figure A2).

**Figure 2:**
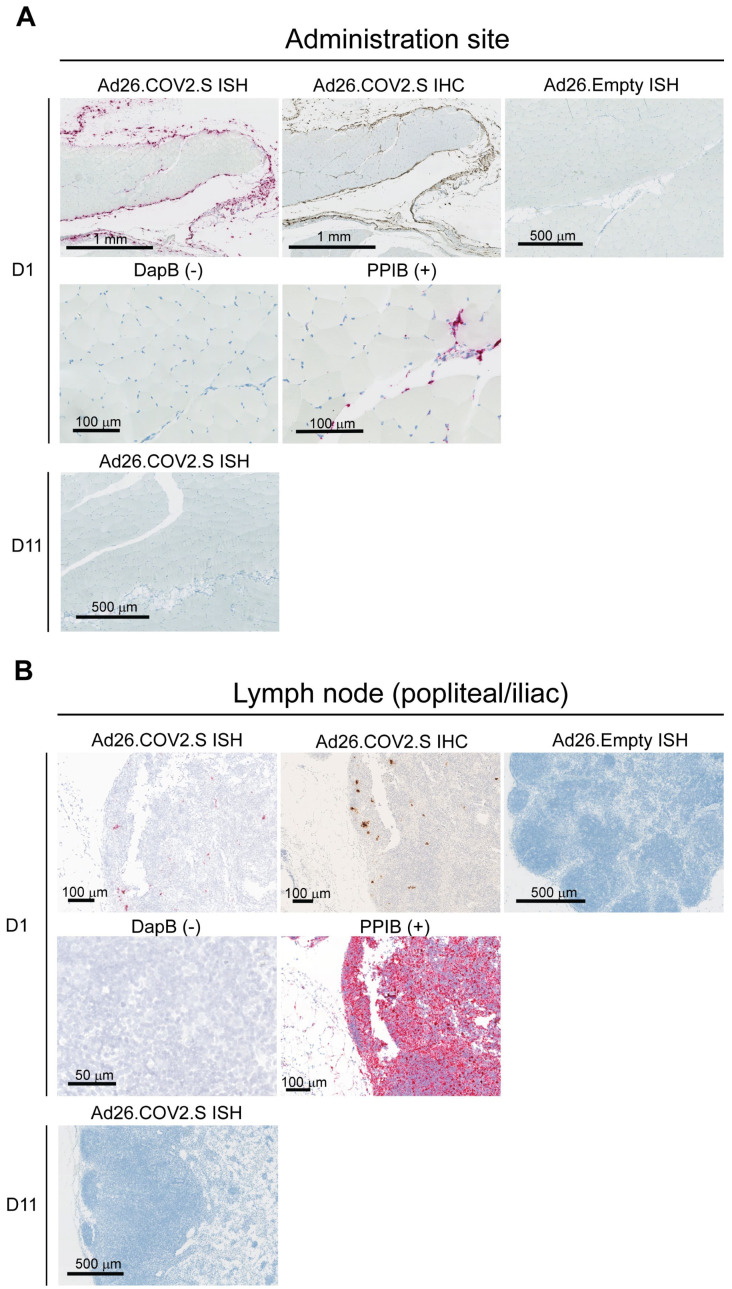
Spike mRNA in situ hybridization (ISH) detected at site of injection and draining lymph nodes 1 day after IM Ad26.COV2.S dosing in rabbits. Anti-SARS-CoV2 S1 staining by immunohistochemistry (IHC) and ISH of spike mRNA at (**A**) the administration site and in (**B**) lymph nodes from rabbits (*n* = 4 per group) at Day 1 after dosing with Ad26.COV2.S (positive) or Ad26.Empty (negative) or ISH on Day 11 after dosing with Ad26.COV2.S (negative). The bacterial gene dihydrodipicolinate reductase (dapB) (negative control) and housekeeping gene cyclosporine-binding protein peptidylpropyl isomerase B (PPIB) (positive control) are shown in the same tissues. In brown (IHC) are the positive areas for the target protein and in purple/red are the positive areas for the target mRNA (ISH). The black bar represents the magnification of the image.

As an alternative method to identify vector-transduced cells, S mRNA was investigated through in situ hybridization (ISH) in Ad26.COV2.S-immunized animals. S mRNA was detected at the site of administration (Figure 2A) and draining lymph nodes (Figure 2B) 1 day after dosing with Ad26.COV2.S consistent with the S-protein expression as detected by IHC. A comparable morphology of positive cells was observed across the two techniques. No S mRNA was detected in any of the tissues at Day 11 after immunization, similar to the S-protein detection through IHC, suggesting the clearance of transduced cells (Figure 2).

### 3.2. S protein Was Detectable in Plasma/Serum after IM Administration of Ad26.COV2.S and Other S-Protein-Encoding Ad26-Based Vectors in Rabbits, but Not in Platelet-Rich Cell Fraction

**Figure 3:**
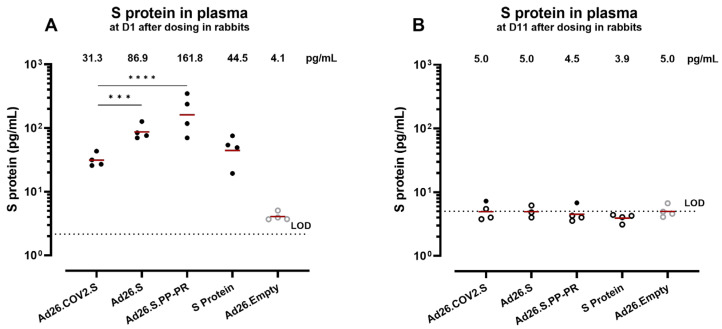
Concentration of S protein in plasma after administration of S protein or Ad26-based vectors encoding the S protein in rabbits. Plasma from rabbits (*n* = 4 per group/timepoint) dosed with Ad26.COV2.S, Ad26.S, Ad26.S.PP-PR, recombinant S protein COR201225 + aluminum hydroxide (Al(OH)_3_), or Ad26.Empty was analyzed at (**A**) Day 1 and (**B**) Day 11 after dosing. Symbols in (**A**) represent the mean response per animal of 2 independent assays. A comparison of the S-protein concentration induced by Ad26.COV2.S and Ad26.S or Ad26.S.PP-PR was performed using a Tobit model with a Bonferroni adjustment for multiple comparisons, *** *p* = 0.0006, **** *p* < 0.0001. The dotted line represents the lower limit of detection (LOD) of the assay based on the standard curve. The background is defined by responses measured after dosing with Ad26.Empty, which does not include or encode SARS-CoV-2 S. Open symbols represent values below the 95th percentile of the Ad26.Empty group. The geometric mean is represented with a red line.

We compared S-protein levels in the blood (both in plasma and in cell fraction) of rabbits dosed IM with different S-encoding Ad26-based vaccines, a subunit S-protein vaccine, or Ad26.Empty (Figure 1A). the S protein was detected in plasma sampled 1 day after administration of the Ad26 vectors encoding the S protein or of the subunit S protein vaccine (Figure 3A). There were significantly lower S-protein levels in Ad26.COV2.S- dosed animals (group geomean 31 pg/mL ± 1) compared with animals receiving Ad26.S (group geomean 87 pg/mL ± 1; *p* = 0.0006, Tobit model with Bonferroni adjustment) or Ad26.S.PP-PR (group geomean 162 pg/mL ± 2; *p* < 0.0001 Tobit model with Bonferroni adjustment). On Day 11 after administration, the S-protein levels in the plasma of all groups were back to background levels (Figure 3B).

**Figure A3:**
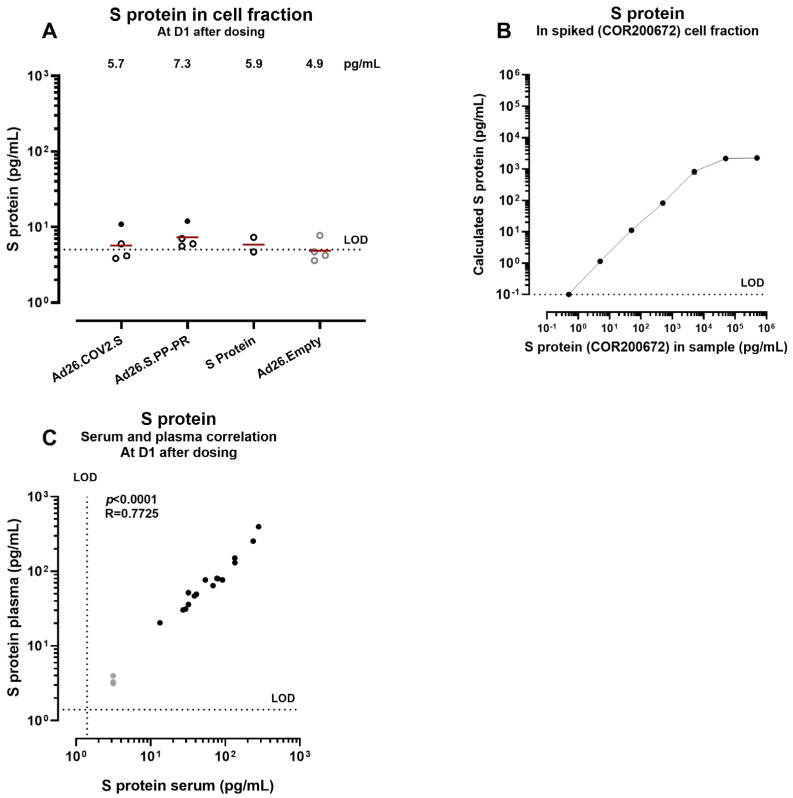
S protein concentration in cellular fraction after dosing with S protein or Ad26-based vectors encoding S protein in rabbits and correlation of S protein concentration in plasma and serum. (**A**) Blood-derived cell fraction was analyzed at Day 1. The background is defined by responses measured after dosing with Ad26.Empty, which does not include or encode SARS-CoV-2 S. Open symbols represent values below the 95th percentile of the Ad26.Empty group. The geometric mean response per group is indicated with a red line and the numbers above the graph. (**B**) Blood-derived cell fraction was isolated before dosing and was spiked with purified S protein COR200672 at increasing concentrations. (**C**) Correlation of S protein concentration in plasma and serum. Black symbols correspond to the samples from rabbits dosed with Ad26.COV2.S, Ad26.S, Ad26.S.PP-PR, or recombinant S protein COR201225 + Al(OH)_3_. Gray symbols correspond to the samples from rabbits (*n* = 4 per group) dosed with Ad26.Empty analyzed at Day 1. Spearman correlation coefficient (R) and *p*-value were calculated for the correlation analysis. The dotted lines represent the lower limit of detection (LOD) of the assays based on the standard curves.

In contrast, the S-protein levels in the blood-derived cell fraction collected 1 day postdosing were in the range of the background measured in the control animals dosed with Ad26.Empty (Figure A3A). We confirmed that cell lysis buffer does not interfere with the S-protein detection assay by spiking purified S protein into the blood-derived cell fraction of naïve rabbits prior to cell lysis (Figure A3B).

The S protein was also detectable in serum and the levels were comparable to the levels detected in plasma (Figure A3C), suggesting that the blood sample preparation method had no major influence on S-protein detection.

### 3.3. Circulating S Protein Presents Similar Expression Kinetics but Different Magnitude of Expression and Subunit Composition after IM Administration of S-Encoding mRNA Compared with Ad26.COV2.S

**Figure A4:**
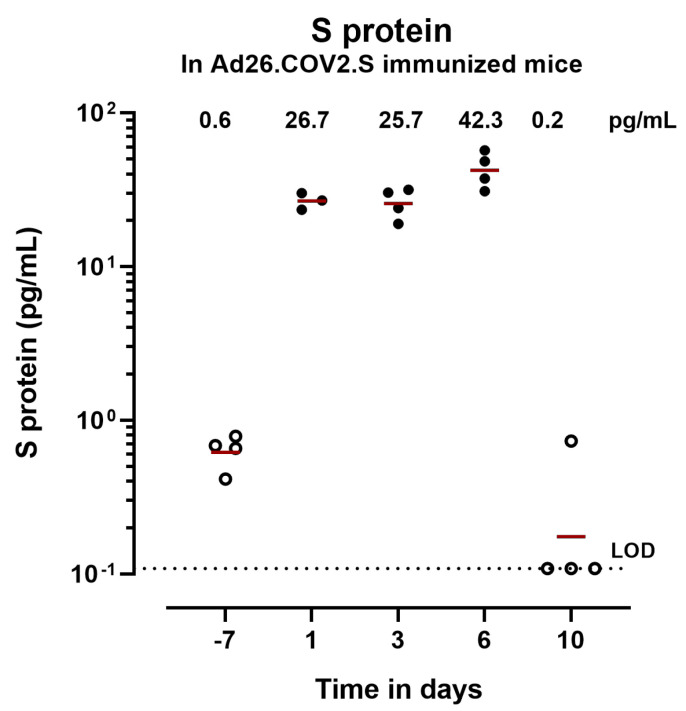
S protein concentration in serum of mice dosed with Ad26.COV2.S. BALB/c mice (4 mice/group, 1 group/timepoint) were dosed with 1 × 10^9^ vp/mouse and S protein was measured in serum at different timepoints (1 symbol/mouse). The dotted line represents the lower LOD of the assay based on the standard curve. Open symbols represent values below the 95th percentile of the predose samples. The geometric mean is represented with a red line.

To follow up on the potential role of the circulating S protein in the development of VITT and make a cross-platform comparison, we assessed the level of S protein after IM administration of S-encoding mRNA vaccines or Ad26.COV2.S in mouse and human serum samples. First, S-protein expression kinetics were determined in the serum of mice dosed with 1 × 10^9^ vp of Ad26.COV2.S (Figure A4). S-protein levels were detected at Day 1 (26.7 pg/mL), remained detectable until Day 6, and decreased to background levels by Day 10 after dosing.

**Figure 4:**
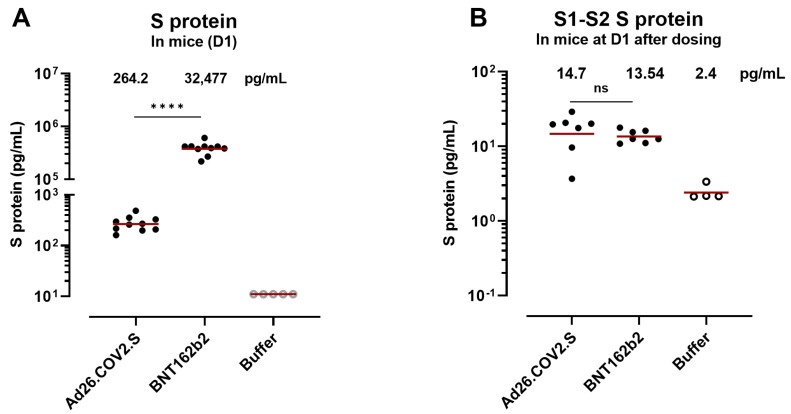
S-protein concentration in mouse serum after Ad26.COV2.S or S-encoding mRNA vaccination. (**A**) S protein was measured in mouse serum at 24 h after dosing with Ad26.COV2.S or BNT162b2. Open symbols represent values below the 95th percentile of the buffer (negative control) samples. (**B**) S1–S2 protein was measured at 24 h after dosing in serum. Comparisons of the S protein concentration and the S1–S2 concentration were performed using a non-parametric *t*-test (Mann–Whitney test), **** *p* < 0.0001, ns, not significant. The geometric mean is represented with a red line.

To compare the S-protein levels after Ad26.COV2.S with an mRNA-based vaccine platform expressing an S protein containing a wild-type furin cleavage site, we dosed mice with 1 × 10^10^ vp of Ad26.COV2.S/mouse (1/5 human dose), 6 μg of BNT162b2 (1/5 human dose), or saline buffer. The S-protein levels in Figure A4 were relatively low, so mice were dosed with a higher amount of vp in this experiment to be able to detect differences with the mRNA vaccine and use the serum for a less sensitive assay (S1–S2 protein). Serum was collected 24 h after dosing to determine S-protein levels were more than 100-fold higher in mice dosed with BNT162b2 (group geomean 32,477 pg/mL ± 1) compared with mice dosed with Ad26.COV2.S (group geomean 264 pg/mL ± 1) (*p* < 0.0001, Mann–Whitney test) 1 day after dosing (Figure 4A). To determine whether the S protein detected in circulation consists of the S1 subunit only or an S protein containing the S1 and S2 domains (S1–S2 protein), serum samples from Day 1 after dosing were evaluated for S protein containing S1–S2 protein (Figure 4B). The S1–S2 protein levels were similar between Ad26.COV2.S and BNT162b2 (not significant, Mann–Whitney test), suggesting a lower degree of S1 shedding for the Ad26-based vaccine.

**Figure A5:**
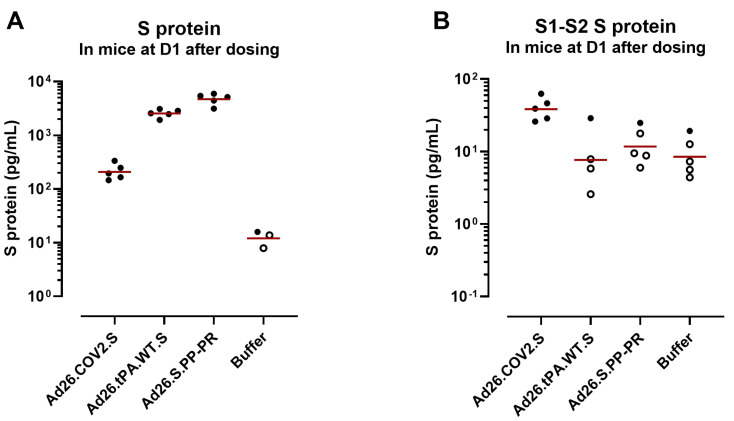
S protein and S1/S2 protein levels in mice immunized with Ad26 constructs encoding different S protein variants. Mice were immunized with 10^10^ vp of Ad26.COV2.S, Ad26.tPA.WT.S, Ad26.S.PP-PR or saline buffer. (**A**) S protein and (**B**) S1–S2 protein were measured at 24 h after dosing in serum. Open symbols represent values below the 95th percentile of the buffer control samples. The geometric mean is represented with a red line.

To determine whether the differences observed in the S1 shedding between Ad26.COV2.S and BNT162b2 were due to differences in the S furin cleavage site, we dosed mice with 1 × 10^10^ vp of Ad26.COV2.S, Ad26 encoding an S protein similar to that of BNT162b2 (Ad26.S.PP-PR), Ad26 encoding an S protein similar to that of ChAdOx1 (Ad26.tPA.WT.S), or saline buffer. Ad26.S.PP-PR and Ad26.tPA.WT.S both encode an S protein containing a wild-type furin cleavage site (Figure A1). Serum was collected at 24 h postdosing and the S protein (as measured by an S1 antibody) and S1–S2 protein levels were measured (Figure A5). In the Ad26.tPA.WT.S and Ad26.S.PP-PR groups, the S-protein levels were higher compared with Ad26.COV2.S while the S1–S2 protein levels were lower, suggesting that the degree of S1 shedding is dependent on the encoded S furin cleavage site.

**Figure 5:**
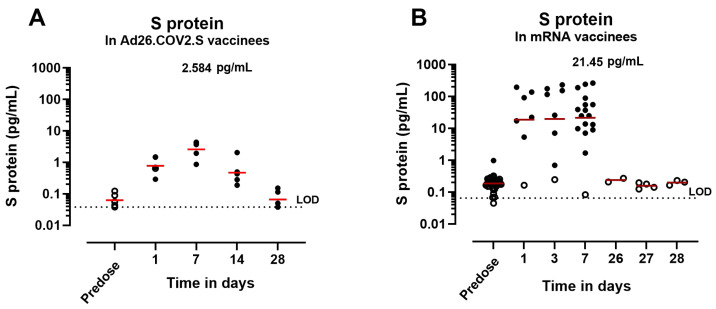
S-protein concentration in human serum after Ad26.COV2.S or S-encoding mRNA vaccination. S-protein expression kinetics in serum from (**A**) five Ad26.COV2.S vaccinees or (**B**) from mRNA vaccinees at different timepoints. Open symbols represent values below the 95th percentile of the predose samples. The geometric mean is represented with a red line and, at Day 7 after dosing, is represented in pg/mL. The dotted lines represent the lower limit of detection (LOD) of the assay based on the standard curve.

Next, S-protein expression kinetics were determined in in human sera from participants who received a COVID-19 vaccine and were seronegative for SARS-CoV-2 before vaccination. Vaccinees received an IM dose of 5 × 10^10^ vp of Ad26.COV2.S (approved human dose) (Figure 5A) or mRNA (30 μg BNT162b2 or 100 μg mRNA-1273, approved human dose) (Figure 5B). The S-protein expression presented similar kinetics between the two groups, with a peak in expression between 3 and 7 days postadministration. At 7 days after Ad26.COV2.S vaccination (group geomean 2.6 pg/mL ± 1.9) the levels were 7.3-fold lower than 7 days after mRNA vaccination (group geomean 21.45 pg/mL ± 7.122). Overall, the levels of the S protein were approximately 10-fold lower in Ad26.COV2.S vaccinees compared with mRNA vaccinees across timepoints. Due to the limited sensitivity of the assay, S1–S2 protein levels could not be determined in human serum. However, higher levels of S1 shedding are expected in mRNA vaccinees compared with Ad26.COV2.S due to the presence of a wild-type furin cleavage site in the S protein, consistent with the results observed in mice. 

### 3.4. Anti-Spike Antibodies Interfere with S-Protein Detection in Serum after IM Administration of Ad26.COV2.S in NHPs and Humans

**Figure 6:**
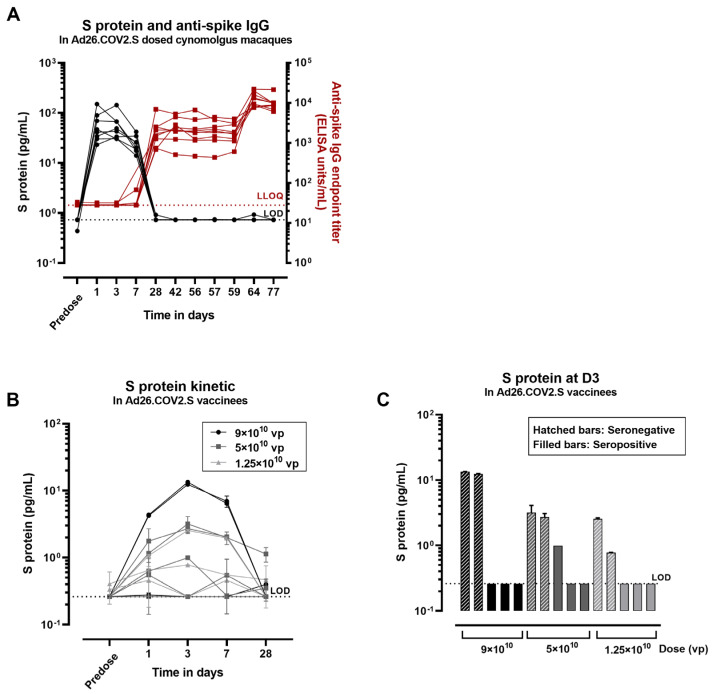
Concentration of S protein in serum after dosing with Ad26.COV2.S in macaques and humans. (**A**) Macaque serum samples were analyzed for S-protein detection and for anti-spike immunoglobulin G (IgG) antibody titers. Black symbols correspond to S concentration expressed in pg/mL and red symbols correspond to anti-spike IgG titers expressed as endpoint titer ELISA (1 symbol/animal). The black dotted line represents the lower limit of detection (LOD) of the S assay based on the standard curve. The red dotted line corresponds to the lower limit of quantification of the anti-spike assay (LLOQ). (**B**) The concentration of S protein was measured in Ad26.COV2.S vaccinees at different timepoints before and after dosing. (**C**) The concentration of the S protein was measured in serum from seropositive vaccinees (containing anti-spike neutralizing antibodies) or seronegative vaccinees 3 days after dosing with Ad26.COV.2.S. The black dotted line represents the lower limit of detection (LOD) of the assay based on the standard curve. The error bars represent the standard deviation of 2 technical replicates in (**B**,**C**).

To assess whether the decrease in S protein to background levels after Ad26.COV2.S immunization is due to the interference of anti-spike antibodies with the detection assay or due to a decrease in transgene expression alone, we analyzed samples from a study in cynomolgus macaques (*n* = 8). The macaques received two full human doses of 5 × 10^10^ vp/animal of Ad26.COV2.S at Days 0 and 56, and serum samples were evaluated for S protein and anti-spike antibodies over time (Figure 6A). The S-protein concentration peaked at Day 1 after the first dose (group mean 60 pg/mL ± 41) and was still detectable at Day 7 (group mean 25 pg/mL ± 9). A decrease to background levels in 7/8 animals was observed at Day 28 after the first dose. The decrease in detectable S-protein levels coincides with the first detection of anti-spike IgG antibody titers on Day 28 (group mean 2208 ELISA units/mL ± 1879). The anti-spike antibody titers remain constant at subsequent timepoints and further increase at Day 64 (Day 8 after the second dose; group mean 13,203 ELISA units/mL ± 5324). In contrast, the S protein was not detectable in serum at any timepoint after the second Ad26.COV2.S dose where, according to primary exposure in naïve animals, S expression would be expected.

**Figure A6:**
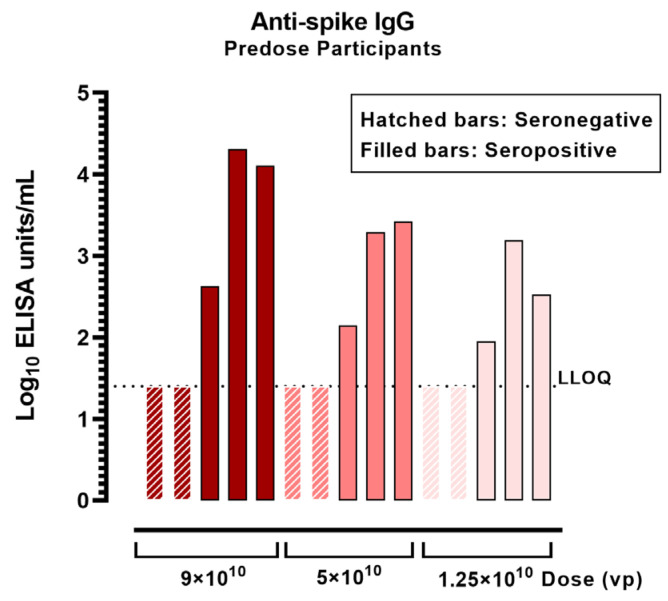
Anti-spike IgG titers in serum before dosing with Ad26.COV2.S in humans. S-specific IgG titers were measured in serum from predose sampling. The dotted line represents the LLOQ of the assay. Each bar corresponds to a different individual. Hatched bars correspond to seronegative individuals (below LLOQ) and filled bars correspond to seropositive individuals (above LLOQ).

To assess S-protein levels in anti-spike antibody seropositive human trial participants, sera from Ad26.COV2.S vaccinees from a dose range study (COV3003) were analyzed. This study was conducted later during the pandemic; therefore, 9 out of 15 participants were anti-spike seropositive at baseline. The S-protein concentration was determined in serum of vaccinees that received an IM dose of 9 × 10^10^ vp, 5 × 10^10^ vp, or 1.25 × 10^10^ vp of Ad26.COV.2.S. Anti-spike antibody levels were measured in the same vaccinees before dosing. In baseline seronegative individuals, S-protein levels peaked at 3 days after dosing (3 pg/mL ± 4), and remained detectable by Day 7 after dosing (Figure 6B). The S-protein levels decreased to near background levels by 28 days after dosing, similar to the S-protein expression kinetics observed in cynomolgus macaques. In contrast, only 1 out of 9 seropositive individuals showed detectable levels of the S protein at Day 3 (Figure 6C). The magnitude of anti-spike binding titers in the serum of this individual was relatively low (log10 2 ELISA units/mL) (Figure A6).

**Figure A7:**
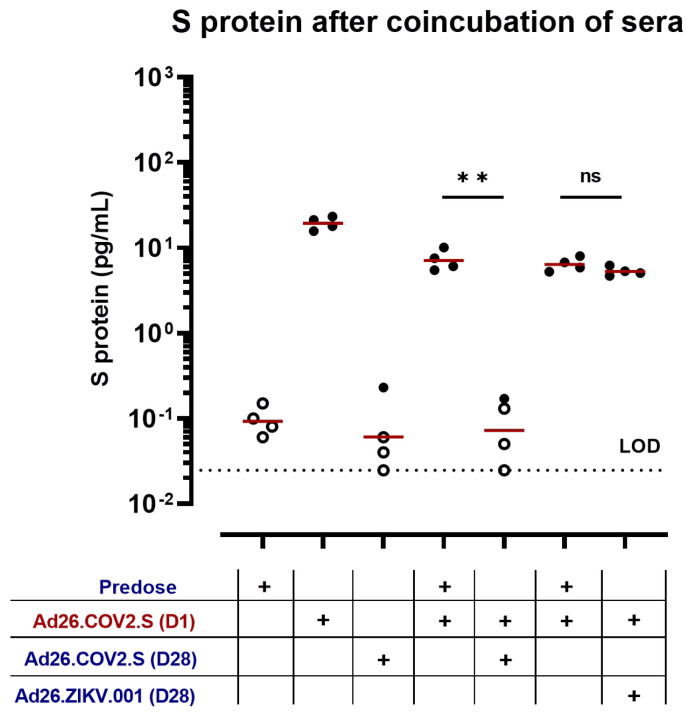
S protein in rabbit serum samples after coincubation with anti-spike antibody containing serum. Spike-containing serum from Day 1 after dosing with Ad26.COV2.S (in red) was coincubated with anti-spike antibody containing serum from 28 days after dosing with Ad26.COV2.S (in blue). Spike-containing serum from Day 1 after dosing (in red) was coincubated with predose serum (from Ad26.COV2.S immunized group—Column 4, or Ad26.ZIKV.001 immunized group—Column 6) or anti-spike antibody deficient serum of rabbits dosed with Ad26.ZIKV.001 (in blue) (negative controls). The dotted line represents the lower limit of detection (LOD) of the assay based on the standard curve. Comparison of the S protein concentration was performed using unpaired *t*-tests with Welch’s correction. The geometric mean is represented with a red line. ** *p* < 0.01.

Since S-protein expression was undetectable upon induction of anti-spike antibodies in NHP and human serum samples, we determined whether anti-spike antibodies interfere with the detection of S protein through the formation of anti-spike IgG–S-protein immune complexes in vitro. Serum containing the S protein was co-incubated with serum containing anti-spike antibody. Rabbit serum (*n* = 4) taken on Day 1 postdosing with 5 × 10^10^ vp of Ad26.COV2.S was co-incubated with predose serum (anti-spike antibody negative) or serum from Day 28 after dosing with 5 × 10^9^ vp/kg of Ad26.COV2.S (anti-spike antibody positive) or Ad26.ZIKV.001 (anti-spike antibody negative) from a different study [45] (Figure A7). The S-protein readouts after co-incubation with Day 28 serum (group mean 0.1 pg/mL ± 0.1) were significantly lower than the S-protein readouts after co-incubation with predose serum (group mean 7 pg/mL ± 2) (*p* = 0.001, unpaired *t*-test) (Figure A7). As a control, S protein containing serum from Day 1 after dosing with Ad26.COV2.S was co-incubated with serum from Day 28 after dosing with Ad26.ZIKV.001. the S-protein readouts were comparable between samples co-incubated with predose serum or Ad26.ZIKV.001 immune serum, suggesting that the anti-spike antibodies, and not other components in the serum after Ad26 vaccination, interfere with the S-protein detection assay. 

### 3.5. Low Levels of Circulating S Protein Were Detectable for a Prolonged Period after Ad26.COV2.S Administration in Antibody-Deficient Mice

**Figure 7:**
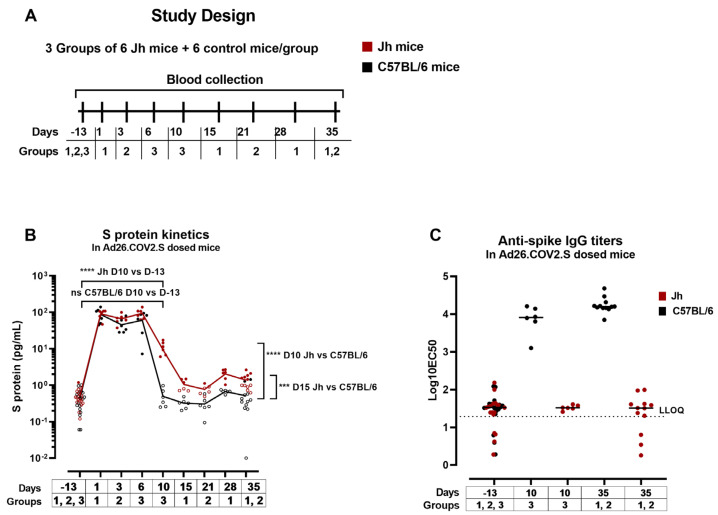
S-protein expression and S-specific IgG titers in antibody-deficient and wild-type mice. (**A**) Antibody-deficient mice (Jh) or control mice (C57BL/6) were dosed with 5 × 10^9^ vp/mouse of Ad26.COV2.S (Groups 1–3). (**B**) Expression of S protein was measured in serum at different timepoints. The open symbols represent the values below the 95th percentile of the predose values. The red (Jh mice) and black (control mice) lines represent the trend of the mean S-protein expression across timepoints. *** *p* < 0.001, **** *p* < 0.0001. (**C**) S-specific IgG titers were measured in serum from predose sampling, 10 days and 35 days after dosing in 2 enzyme-linked immunosorbent assay (ELISA) runs. The dotted line represents the lower limit of quantification (LLOQ) of the assay. Each symbol (circles, squares, and triangles) corresponds to a different group of mice. Statistical comparisons were performed using paired *t*-tests or unpaired *t*-tests when comparing different mice.

In vaccinees who do not mount sufficiently inactivating anti-spike antibody responses after vaccination, the free circulating S protein might be present for a prolonged period of time and could be an additional factor in the development of VITT. To further investigate the duration of the S-protein expression in an anti-spike antibody free model Jh (C57BL/6NTac-Igh-Jem1Tac) knockout or C57BL/6 (background control), mice were dosed with 5 × 10^9^ vp/mouse of Ad26.COV2.S (Groups 1–3) (Figure 7A). Jh mice carry a deletion of the endogenous murine J segments of the Ig heavy chain locus and therefore contain no mature B cells and produce no antibodies [46].

The S protein was detected at Days 1, 3, and 6 after dosing with Ad26.COV2.S at comparable levels across groups and timepoints (Figure 7B). Ten days after dosing with Ad26.COV2.S, S-protein expression was detectable in Jh mice (group mean 11 pg/mL ± 4) (*p* < 0.0001, paired *t*-test) and not in C57BL/6 mice (group mean 0.5 pg/mL ± 0.3) (ns, paired *t*-test) (Figure 7B). At Day 15 postdosing, the S-protein levels were higher in Jh (1 pg/mL ± 0.4) compared with C57BL/6 mice (0.3 pg/mL ± 0.1) (*p* = 0.0001, unpaired *t*-test). The S protein was detectable at low levels until Day 35 after dosing in the Jh (group mean 1.1 pg/mL ± 0.4), but not in C57BL/6 mice.

Anti-spike IgG antibodies were measured in serum at Day 10 and Day 35 after dosing. Anti-spike antibody levels were higher at Day 35 after dosing compared with Day 10 in C57BL/6 mice (*p* = 0.0135, unpaired *t*-test) and anti-spike antibodies were not detectable in Jh mice at any of the timepoints (Figure 7C). These data suggest the interference of anti-spike antibodies with the S-protein detection or the clearance of the S protein and/or S-expressing cells by anti-spike immune complexes. 

## 4. Discussion

VITT is a rare adverse event characterized by thrombosis in atypical anatomical locations and severe thrombocytopenia. The mechanism behind VITT has not yet been fully elucidated, but it may result from a combination of factors, including the activation of platelets/endothelial cells and the inflammatory signatures induced by the SARS-CoV-2 S protein after vaccination. Here, we assessed the distribution of the S protein and characterized the circulating S protein after Ad26.COV2.S vaccination in animal models. Additionally, we evaluated circulating S-protein levels in clinical samples. 

We detected the S protein and S mRNA at the site of administration (muscle) and in draining lymph nodes 1 day after the dosing of rabbits with Ad26.COV2.S, but not in the blood vessel wall or lining endothelium (arteries/veins). The SARS-CoV-2 S protein has been shown to cause vascular damage in hamsters [30] and has been detected within the thrombus and in the adjacent blood vessel wall in VITT patients with cerebral venous thrombosis [31]. In our studies, we did not detect the S protein in blood vessel walls or observe evidence of endothelial damage in rabbits. However, the effects of the circulating S protein on endothelial cells may still play a role on VITT as published [29,47,48], and may depend on the exposure, kinetics, cellular uptake and signaling dynamics, and subunit composition of the circulating S protein.

Eleven days after dosing with Ad26.COV2.S, we did not detect the S protein by immunohistochemistry or S mRNA by in situ hybridization in any of the rabbit tissues examined. The circulating S protein was not detectable by Day 10/11 in the serum of animal models or by Day 29 in the serum of human vaccinees. Consistent with our results, Stebbings et al. found that the S protein was rapidly undetectable (within Days 7–14) in the circulation of mice dosed intravenously or IM with ChAdOx1 nCoV-19 [49]. This decrease in S-protein detection is likely mainly due to the clearance of the transduced cells and the adenoviral vaccine. Alternatively, anti-spike antibodies may interfere with the detection of the S protein. Indeed, in an antibody-deficient mouse model, we measured low but detectable levels of circulating S protein up to Day 35 after dosing, suggesting partial antibody-mediated clearance of the S protein. This would explain the early timing of induction of VITT and the lower incidence of VITT after a second dose of the vaccine [13], if the S protein plays a role in the process. In alignment with this, the prolonged bioavailability of the S protein may play a role in the development of VITT. In some human cases of VITT, circulating S protein has been detected up to 35 days after vaccination [50]; however, limited data are available from VITT patients. The lack of anti-spike antibody complex formation with the S protein and the persistence of free circulating S protein for a prolonged period of time due to host-specific factors may drive prolonged proinflammatory signatures via S binding and the activation of specific cell types, such as endothelial cells, as previously hypothesized [30].

In our studies, significantly lower levels of the S protein, as detected by an S1 antibody, were observed after Ad26.COV2.S administration compared with the levels observed after administration of an mRNA vaccine in mice and human vaccinees, while the S1–S2 protein presented similar levels across these two vaccine platforms in mice. The mRNA vaccines for COVID-19 (BNT162b2 and mRNA-1273) express an S protein that contains a wild-type furin cleavage site, which has been shown to result in the shedding of the S1 portion of the S protein in the plasma of mRNA-1273 vaccinees, consistent with our results [4]. VITT has been reported after ChAdOx1 vaccination in humans [14]; however, ChAdOx1 encodes a wild-type S that presented similar S1 shedding properties compared with mRNA vaccines in our studies. Therefore, the differences in bioavailability of S1 are unlikely the main triggers of VITT, however, S protein may still contribute to the VITT pathogenesis in combination with other factors.

## 5. Conclusions

Overall, we showed no detection of the S protein in the endothelium or bound to platelets after Ad26.COV2.S vaccination in animal models. We have demonstrated similar kinetics of transient S-protein expression after Ad26.COV2.S vaccination in preclinical models and humans, with a comparable kinetic but lower magnitude as observed after mRNA COVID-19 vaccination. The S subunit composition was different in serum after Ad26.COV2.S compared with mRNA BNT162b2 dosing in preclinical models. This is likely linked to the mutated furin cleavage site of Ad26.COV2.S, which however is not present in ChAdOx1, the other VITT-related vaccine, making it an unlikely trigger of VITT. The observations of the S-protein biodistribution, kinetics, and composition after Ad26.COV2.S vaccination do not provide conclusive evidence for the absence or presence of a direct association of the S protein with the development of VITT. The S protein, in the context of Ad26 vaccination, cannot be excluded as a potential contributing factor in the pathogenesis of VITT in combination with other factors such as the Ad26-related innate immune stimulation, previous infections, genetic predisposition, or pre-existing health conditions that likely influence the development of VITT.

## Data Availability

The data presented in this study are available on request from the corresponding author.
